# Metacognitive monitoring and calibration in the vocal emotion recognition task

**DOI:** 10.3389/fpsyg.2026.1763494

**Published:** 2026-04-22

**Authors:** Rachel-Tzofia Sinvani, Haya Fogel-Grinvald

**Affiliations:** 1 Talpiot Academic College, Holon, Israel; 2 School of Occupational Therapy, Faculty of Medicine, Hebrew University of Jerusalem School of Medicine, Jerusalem, Israel

**Keywords:** calibration, emotion, metacognition, overconfidence, speech

## Abstract

**Background and aims:**

The influence of specific task demands on metacognition in socio-emotional processing, particularly emotion recognition, remains poorly understood. In this study, we aimed to address this gap by examining differences in metacognitive monitoring (MM) using self-rated confidence (SRC), metacognitive accuracy (MA), and objective vocal emotion recognition ability (ERA) across discrete emotions.

**Methods:**

To this end, we used a categorical emotion recognition task. A hundred participants listened and were asked to identify one of six vocal emotions (anger, sadness, surprise, happiness, fear, and neutrality) in eighteen semantically neutral sentences. Immediately following each recognition response, participants provided a retrospective SRC.

**Results:**

A series of one-way repeated-measures ANOVAs revealed a significant main effect of emotion on MM, MA, and ERA. Post-hoc pairwise comparisons indicated no significant differences between specific emotions for MM. Conversely, significant differences between emotions were observed for both MA and ERA, which exhibited a similar pattern of variability. Notably, sadness demonstrated the highest calibration and accuracy, while fear showed the poorest performance on both measures.

**Conclusion:**

Our findings suggest that the differences in MA between emotions are primarily attributed to the accuracy of ERA variations rather than the variations in MM. These results indicate that specific emotional content acts as a distinct task demand, influencing performance and metacognitive outcomes. Future research should explore the underlying mechanisms of MM in emotion recognition by comparing various MA indices across tasks and domains to provide a more comprehensive understanding of MA in the social domain.

## Introduction

Metacognitive monitoring (MM), the ongoing evaluation of one’s cognitive processes (Flavel, 1979), is fundamental for guiding learning behaviors (Efklides, 2009). A critical dimension of this process is metacognitive accuracy (MA) (Ariel et al., 2018), often operationalized as calibration, or the degree of correspondence between subjective confidence and objective performance (Keren, 1991; Schraw, 2009). Discrepancies in calibration manifest as over- or underconfidence, providing essential insights into cognitive self-awareness (Fleming and Lau, 2014; Baranski and Petrusic, 1994). Over the last three decades, research has increasingly focused on the role of stimulus characteristics in shaping metacognitive evaluations, primarily across diverse cognitive tasks (Laursen, 2025; Fiedler et al., 2019; Koriat, 1997); however, a comparable understanding of stimulus-related effects across social–emotional tasks is scarce (Agadzhanyan and Castel, 2024). While recent evidence from visual emotion recognition tasks demonstrates that emotional cues differentially influence both MM and calibration (Bègue et al., 2019; Dentakos et al., 2019), the vocal channel has been largely overlooked (Laukka et al., 2021; Sinvani et al., 2024). To bridge this gap, the current study investigates the effect of different emotion categories on both monitoring levels (MM) and calibration (MA) in the vocal emotion recognition task.

### Metacognitive monitoring (MM)

Metacognition is fundamentally defined as cognition about one’s own cognitive processes, functioning through the dynamic interplay between monitoring and control (Flavel, 1979; Moritz and Lysaker, 2018). Metacognitive monitoring (MM) encompasses the processes by which individuals evaluate and track their ongoing cognitive performance, while control involves using this knowledge to regulate cognition (Fiedler et al., 2019). Indeed, according to the framework established by Nelson and Narens (Nelson, 1990), the output of the MM function serves as the direct input to metacognitive control. By regulating cognitive strategies based on these monitoring outputs, metacognitive control processes manage the costs and benefits associated with judgments and decisions, thereby playing a crucial role in performance optimization (Ackerman and Thompson, 2017; Wixted, 2007; Wixted and Wells, 2017; Wixted, 2020).

Contemporary research indicates that MM can manifest in both global and local metacognition (Seow et al., 2021; Schraw, 1994). Global metacognition refers to a general self-awareness of how a cognitive process operates in one’s own mind. Therefore, global metacognition can be measured using self-reported questionnaires on general ability, comfort, or confidence in a cognitive task (Efklides, 2006; Händel et al., 2020). Conversely, local metacognition refers to online metacognitive processing that is specific to an ongoing task. Accordingly, local judgments are measured item by item. Various methodologies have been employed to assess MM. Perhaps the most common measure, locally or globally, is self-rated confidence (SRC), or one’s confidence in the correctness of a given performance (Fiedler et al., 2019; Nelson, 1990). Elicited by simply asking an individual to provide commentary on their response accuracy (Kelly and Metcalfe, 2011), several studies have come to use SRC, assigned to prospective or retrospective task performance (Dentakos et al., 2019; Kelly and Metcalfe, 2011; Stankov, 2019; Hoogervorst et al., 2024). Interestingly, local-level analysis has revealed greater variability in SRC across measures than in global measures (Agadzhanyan and Castel, 2024; Dentakos et al., 2019). Therefore, given that local SRC is reported on an item-by-item basis, researchers have suggested that certain effects may stem from task demands and the ways these demands influence participants’ responses (Hoogervorst et al., 2024; Katyal and Fleming, 2023; Katyal and Fleming, 2024; Double, 2025; Schwartz, 2024).

### Metacognitive accuracy (MA)

Individuals may overestimate or underestimate their actual performance. This discrepancy underscores the importance of assessing metacognitive accuracy, which enables researchers and clinicians to evaluate cognitive self-awareness (Fleming and Lau, 2014) and design targeted interventions accordingly (Saenz et al., 2019). Hence, beyond monitoring levels, it is critical to assess the *accuracy* of metacognitive judgments. In this vein, SRC and its relation to performance are further used to reflect different aspects of metacognitive accuracy (MA), including both resolution and calibration. *Resolution* is one of the main measures for MA, representing the accuracy-confidence association (Baranski and Petrusic, 1994). Resolution, known as relative accuracy, measures the extent to which people can discriminate whether they answered a particular question accurately, on an item-by-item basis. A strong relationship between performance and confidence in their performance indicates a higher resolution. Notably, the Goodman–Kruskal gamma correlation (Nelson, 1984) is commonly employed to quantify, within subjects, the association between SRC judgments and response accuracy on an item-by-item basis. The resolution has been studied in various domains, including visual perception (Kim and Chong, 2024), comprehension (Hausman et al., 2021), problem solving (Zepeda and Nokes-Malach, 2023), numerosity (Fitzsimmons and Thompson, 2024) and the social domain (Sinvani et al., 2024).

Another aspect of MA is *calibration*. Scholars have defined calibration, or absolute accuracy, as the degree to which an individual’s perceptions of their performance correspond with their actual performance, on average (Keren, 1991). Practically, calibration represents the degree of fit between averaged subjective feelings of certainty or correctness about one’s performance and the averaged accuracy in the task (Schraw, 2009). The more subjective confidence judgments match objective performance on average, the better calibrated the individual is considered (Fleming and Lau, 2014). Mismatches between performance and confidence average scores are defined as poor calibration, which can happen in two directions: overconfidence and underconfidence (Baranski and Petrusic, 1994). *Overconfidence* occurs when subjective confidence judgments exceed objective performance, reflecting a failure to detect errors [e.g., high confidence in incorrect responses (Dentakos et al., 2019)]. In contrast, *underconfidence* occurs when subjective confidence judgments are lower than actual performance, reflecting a failure to detect success [e.g., a lack of confidence in correct responses (Stankov et al., 2012)]. Moreover, focusing on calibration, overconfidence is more pronounced across domains (Stankov, 2019; Stankov and Lee, 2014; Binnendyk and Pennycook, 2024; Sidi et al., 2018; Chatzipanteli et al., 2025).

### MM and MA in emotion recognition ability (ERA)

ERA refers to the capacity to accurately identify emotional states from various expressive cues (Bänziger et al., 2015). Accurate ERA is essential for decoding social cues, which in turn supports relationship quality and everyday social interactions (Connolly et al., 2020; Garcia-Cordero et al., 2021). ERA has been studied through multiple theoretical frameworks, including discrete emotion models (Ekman, 1999), dimensional approaches (Russell, 2003), and constructed perspectives (Barrett, 2017). While these frameworks differ in their conceptualization of emotional organization, they all acknowledge that ERA can be assessed across multiple sensory modalities. Despite its automatic nature, substantial individual differences in ERA accuracy persist across modalities (Hayes et al., 2020; Lin et al., 2020).

In the context of ERA, MM is theorized to guide social skills by allowing individuals to learn from errors and seek additional information when interpreting ambiguous expressions, thereby maximizing adaptive responses (Kelly and Metcalfe, 2011; Mitsea et al., 2023). This framework suggests that impaired ERA may stem not only from recognition deficits but also from insufficient MA [i.e., the capacity to evaluate MM accuracy (Katyal and Fleming, 2023; Hoven et al., 2019; Elfenbein and MacCann, 2017)]. Supporting this, evidence shows that ERA competence can be improved through targeted interventions, illustrating the role of metacognition in guiding plasticity (Mitsea et al., 2023).

Over the past two decades, MM has become a central framework for examining how individuals evaluate their own perceptual or cognitive judgments in emotional contexts (Garcia-Cordero et al., 2021; Kelly, 2013; Valenti et al., 2025). Kelly and Metcalfe (Kelly and Metcalfe, 2011) established this logic to visual emotion cues, revealing that retrospective SRC judgments in facial and eye expressions were predictive of task accuracy, even when global prospective confidence was not. However, their work did not account for emotion-related differences in SRC. Bègue et al. (2019) developed a novel paradigm to directly evaluate behavioral parameters and neural substrates underlying metacognition in facial emotion recognition and visual size-perception tasks. By examining varying levels of emotional intensity in anger and happiness, they demonstrated that SRC remained high, comparable to the ERA performance, regardless of stimulus intensity. However, the results are inconsistent across channel modalities. In this vein, a few studies have demonstrated that emotion cues such as valence, arousal, and intensity did modulate MM. For example, Agadzhanyan and Castel (2024) showed that valence text stimuli affect retrospective MM judgments, while Troiano et al. (2021) showed further effects focusing on emotion intensity. Moreover, Convertino et al. (2025) reported similar modulations in tactile experiences.

Despite this growing body of evidence across multiple sensory channels, little is known about how these processes unfold in the vocal domain, a channel central to human communication and emotional expression (Bryant, 2022). Research on emotion in voice has traditionally focused on the clarity and perceptual robustness of acoustic cues rather than the metacognitive awareness of them. Pioneering gating studies, for instance, employed confidence ratings to determine the “identification points” at which listeners reached stable emotion recognition (Pell and Kotz, 2011; Jiang et al., 2015; Cornew et al., 2010). Findings showed that confidence emerged earlier for emotions such as anger and sadness than for fear, indicating variability in perceptual certainty across categories (Pell and Kotz, 2011; Jiang et al., 2015). Similarly, the validation of the Geneva Multimodal Emotion Portrayal (GEMEP) corpus relied on recognition accuracy, inter-rater agreement, and continuous ratings of believability and intensity as implicit indicators of signal clarity, not as measures of explicit metacognitive performance (Bänziger et al., 2009; Bänziger et al., 2012).

These previous studies thus treated confidence as an indirect marker of sensory reliability (Zhu, 2013; Pell et al., 2026; Villani et al., 2025). Only recently has the field further approached confidence in speech-based emotion recognition as a metacognitive construct, opening the way for current research to examine how listeners monitor and evaluate their own accuracy in vocal emotion recognition. Lausen and Hammerschmidt (2020) investigated how emotion category and prosodic parameters predict confidence and accuracy across seven emotions (anger, disgust, fear, happiness, neutral, sadness, and surprise) using both speech-embedded prosody and affect bursts in German. Participants rated their confidence on a 7-point scale (1 = not at all confident, 7 = extremely confident) after categorization. They illustrated stimulus characteristics effect on SRC, showing that listeners were more confident when identifying affect bursts than speech-embedded prosody, and that angry and neutral emotions were identified most accurately. Moreover, confidence varied by emotion category, demonstrating emotion-related differences in SRC within the vocal ERA (Lausen and Hammerschmidt, 2020).

While initial research confirms a systematic link between SRC and accuracy in ERA across various channels, explicit investigations into MA remain relatively scarce (Laukka et al., 2021; Sinvani et al., 2024; Kelly and Metcalfe, 2011). Notably, these studies largely examined aggregate performance and did not systematically explore whether MA varies with emotion cues. Furthermore, given that the literature has focused almost exclusively on the resolution index to measure MA in ERA, the calibration index has been largely unapplied. To our knowledge, only two studies implement calibration in ERA based on the visual channel. Specifically, Dentakos et al. (2019) examined resolution and calibration across diverse cognitive tasks, as well as in facial ERA. They demonstrated that calibration accuracy persists across diverse domains (e.g., finance, general knowledge, and visual ERA). Interestingly, Bègue et al. (2019) observed that in visual ERA specifically, confidence does not drop with ambiguous targets, unlike in other perceptual tasks. Finally, both investigations reveal a functional dissociation between resolution and calibration components, as the ability to discriminate between correct and incorrect trials (i.e., resolution) was found to be task-dependent and significantly lower in visual ERA than in other cognitive fields, while the bias in confidence (i.e., calibration) shows a domain-general trend. Notably, both investigations were consistent in their findings regarding the overconfidence trend across emotional cues (Bègue et al., 2019; Dentakos et al., 2019). Based on their findings, one would argue that when tasks are diverse, resolution might be domain-specific, supporting previous claims that calibration and resolution are conceptually distinct and empirically separable indicators of MA (Ackerman and Thompson, 2017; Ais et al., 2016). This distinction underscores the importance of investigating calibration as a distinct and essential dimension of MA in ERA.

### The present study

While the majority of research in ERA has traditionally focused on the visual channel, our understanding of metacognitive processes in the vocal modality remains limited. Furthermore, existing studies in the vocal domain have predominantly focused on resolution (discrimination), leaving a significant gap regarding calibration (over/underconfidence). Given the distinct theoretical and functional contributions of each metacognitive index, it is unclear whether the consistent emotion-related effects on calibration observed in visual tasks emerge similarly in the vocal modality, where acoustic cues provide fundamentally different diagnostic information. The current study addresses this gap by investigating the impact of specific emotional categories on both metacognitive monitoring (MM) and metacognitive accuracy (MA) within the vocal domain. We followed the approach used by Lausen and Hammerschmidt (2020) and by Laukka et al. (2021) so that we could directly compare our results with previous studies and investigate how specific emotion categories affect metacognitive judgments. In particular, we asked whether SRC and calibration differ significantly across discrete emotions. Since the calibration index integrates SRC and accuracy, we hypothesized that both SRC and calibration would vary with emotion, reflecting differences in the recognizability of specific vocal expressions.

## Materials and methods

The dataset for the current investigation was used in previous studies (Sinvani et al., 2024; Sinvani and Fogel-Grinvald, 2024), in which the authors provided full methodological details, including the sample descriptions and techniques used to elicit, record, and judge vocal emotional expressions.

### Stimuli

We used vocal stimuli originally designed in Hebrew (Sinvani and Sapir, 2022) in the form of repeated lexical sentences, meaning “Oh really, I cannot believe it” [/Ma/Be/e/met/A/ni/lo/Ma/a/mi/na/], reflecting discrete emotions (anger, sadness, happiness, fear, surprise, and neutrality) as well as neutral expression. In this way, we intended to focus on emotional prosodic cues separately from semantic cues. Earlier studies have discussed such methodologies (Sinvani and Sapir, 2022; Paulmann and Kotz, 2008), with particular recommendations in the diagnostic analysis of nonverbal accuracy 2 (DANVA-2), emphasizing the independent role of prosodic cues in emotion recognition (Nowicki and Duke, 2008). Vocal stimuli were recorded by 30 actors × six emotions (anger, happiness, sadness, fear, surprise, and neutrality) × one sentence, finally resulting in 180 emotional utterances.

### Participants

A sample of 100 participants (50 men and 50 women) aged 20–35 years (*M*_age_ = 24.37, *SD* = 2.28) enrolled in the study. All were native Hebrew speakers, undergraduate or graduate students at the University of Haifa. No history of speech, language, or hearing disorders or psychiatric illnesses was detected. Normal hearing of all participants was verified by a standard audiological screening procedure (Koerner and Zhang, 2018).

### Procedure

A sample of 30 professional (eight men, seven women) and nonprofessional actors (eight men, seven women), *M_age_* = 25.07, *SD* = 2.29, was recruited for the recording stage. The speech recordings were obtained in a soundproof booth using a head-mounted condenser microphone (AKG C410) positioned 10 cm and 45°–50° from the left oral angle. The signal was pre-amplified, low-pass filtered at 9.8 kHz, and digitized to a computer hard disk at a sampling rate of 22 kHz (Stipancic and Tjaden, 2022) using Cool Edit Pro (Version 2.0). All audio clips were scanned for adequate amplitude and the absence of missing data (i.e., speakers produced all utterances). The experimental stage was conducted in a soundproof room at the Interdisciplinary Clinics Center at the University of Haifa. The procedure began with participants receiving a detailed description of the experimental tasks and protocol. Subsequently, participants provided informed consent, completed a short demographic questionnaire, and underwent a brief hearing test.

The experimenter presented each vocal target to the participant through Sennheiser HD 448 headphones, which were connected to a Dell OptiPlex 780 SFF desktop PC. Emotional recognition accuracy (ERA) was tested by having participants assign categorical emotion judgments to each of the 18 vocal targets, with three targets for each emotion category presented in a randomized order. These categorical judgments were recorded manually on paper with a pencil by circling the chosen emotion: happiness, anger, sadness, fear, surprise, or neutrality. Self-reported confidence (SRC) was measured retrospectively for each recognition response using a Likert-type scale from 0 (not at all confident) to 6 (extremely confident). The experiment consisted of ten sessions, each with ten participants, and lasted approximately 10 min.

All participants completed all 18 trials, resulting in a complete dataset with no missing values. Therefore, no data imputation or averaging procedures were necessary. Mean accuracy in ERA scores and mean normalized SRC scores (obtained by dividing each original score by six) were computed, resulting in two measures on a unified scale from 0 to 1 for each emotion. The calibration index was then calculated, with values ranging from 0 (miscalibration) to 1 (perfect calibration).


Calibration Index=1−∣Accuracy−normalizedSRC∣

Whereas previous studies have often used the bias index, defined as the difference between participants’ average confidence ratings and actual accuracy (Jackson and Kleitman, 2014), with a scale ranging from −1 (underconfidence) to +1 (overconfidence), we adopted the rationale proposed by Dentakos et al. (2019). Specifically, the bidirectional nature of the bias index limits its applicability in correlational analyses involving unidirectional individual-difference variables. To overcome this limitation, we computed a calibration index that captures the absolute magnitude of the differences between SRC and accuracy in ERA, regardless of direction (Stanovich et al., 2016). Consistent with Stanovich et al. (2016), this score was then reverse-coded to produce a calibration accuracy index, where higher scores indicate greater resistance to overconfidence, which is, in other words, better calibration. This approach enables a meaningful comparison with traditional gamma correlation analyses, as the resolution index, which has higher values, also reflects higher metacognitive sensitivity.

### Ethics statement

This study has Haifa University Human Research Ethics Committee approval (HU Ref No: 252/16). All participants gave their written formal consent to participate in the study, and they were paid 20 NIS for their participation.

### Data analysis

Statistical analysis was performed using IBM SPSS Statistics (SPSS) version 29.0. Mean accuracy in ERA scores and mean normalized SRC scores (obtained by dividing each original score by six) were computed, resulting in two measures on a unified scale from 0 to 1 for each emotion. The calibration index was then calculated, with values ranging from 0 (miscalibration) to 1 (perfect calibration).

Three one-way repeated-measure ANOVAs were used to assess the differences in the calibration index, ERA, and SRC by emotion category. Pairwise comparisons with Bonferroni corrections were used. A *p* < 0.05 was considered statistically significant. Effect sizes were estimated using partial *η* (Efklides, 2009). Values of 0.01 indicate a small effect, 0.06 a medium effect, and 0.14 or higher a large effect (Cohen, 1988). The required sample size for the present study was determined using G*Power 3.1.9.7 software. The minimum sample for the one-way repeated-measures ANOVA was calculated. Assuming a moderate effect size (*f* = 0.25), an *α* level of 0.05, and a power of 0.95, the required sample size was estimated at approximately 59 decoders.

## Results

To investigate the differences between emotions in SRC, a one-way repeated-measures ANOVA was conducted. The assumption of sphericity was met, as assessed by Mauchly’s test of sphericity, *χ*^2^(14) = 23.10, *p* = 0.059. The analysis revealed a significant main effect of the emotion category with a small effect size (*F*_(5,495)_ = 2.99, *p* = 0.011; *η*^2^ = 0.029; see Table 1). However, pairwise comparisons with Bonferroni correction did not reveal any significant differences in SRC across the six emotions. This pattern suggests that while there is overall variability in confidence across emotions, specific emotion contrasts do not reach statistical significance after correction for multiple comparisons, possibly due to the conservative nature of the Bonferroni adjustment.

**Table 1:** Mean and SD for SRC, calibration, and accuracy in ERA by emotions.

Emotion	SRC		Calibration^a^		Accuracy in ERA	
	M	(SD)	M	(SD)	M	(SD)
Fear	0.34	(0.27)	0.74	(0.14)	0.57	(0.26)
Anger	0.42	(0.30)	0.75	(0.15)	0.63	(0.27)
Happiness	0.58	(0.30)	0.78	(0.13)	0.71	(0.22)
Neutrality	0.62	(0.32)	0.73	(0.16)	0.73	(0.22)
Sadness	0.77	(0.27)	0.78	(0.16)	0.81	(0.16)
Surprise	0.51	(0.29)	0.76	(0.14)	0.64	(0.21)

SRC, self-rated confidence; ERA, emotion recognition ability.

a A higher score indicates a larger discrepancy between accuracy in ERA and SRC.

To examine differences in calibration across emotions, a one-way repeated-measures ANOVA was conducted. The assumption of sphericity was not met, as assessed by Mauchly’s test of sphericity, *χ*^2^(14) = 38.80, *p* < 0.001. Epsilon (*ε*) was 0.876, as calculated according to (Greenhouse and Geisser, 1959), and was used to correct the one-way repeated measures ANOVA. As hypothesized, the analysis revealed a significant main effect of the emotion category with a large effect size (*F* (4.38, 433.74) = 15.46, *p* < 0.001, *η*^2^ = 0.14; see Table 1). Pairwise comparisons with Bonferroni corrections revealed a significant difference in calibration for sadness, which showed the highest calibration among anger, fear, and surprise (*p* < 0.001). The calibration in sadness was also significantly higher than for happiness (*p* = 0.002). The calibration in fear was significantly lower compared to happiness and neutrality (*p* < 0.001).

**Figure 1:**
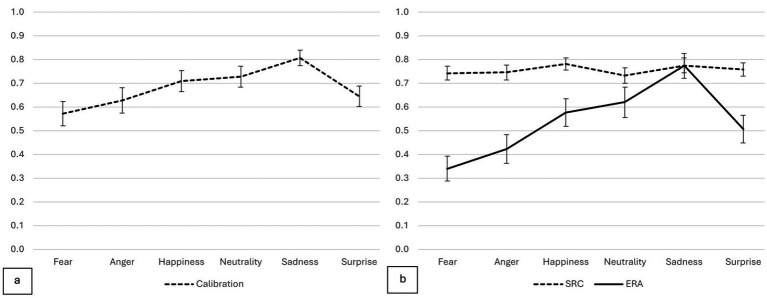
**(a)** Displays the calibration index for each emotion, highlighting the degree of alignment between confidence and accuracy. **(b)** Illustrates the differences between emotions for both emotional recognition accuracy (ERA), represented by a solid line, and self-reported confidence (SRC), shown with a dashed line.

To determine whether ERA differs by various emotions, a one-way repeated-measure ANOVA was conducted. The assumption of sphericity was not met, as assessed by Mauchly’s test, *χ*^2^(14) = 22.69, *p* = 0.066. Similarly to the previous analysis, a significant main effect was found for emotion category with a large effect size (*F*_(5,495)_ = 29.53, *p* < 0.001; *η*^2^ = 0.23; see Table 1). Pairwise comparisons with Bonferroni corrections revealed that the accuracy of sadness was significantly higher compared to all other emotions (*p* < 0.001). Fear had a significantly lower accuracy compared to neutrality, surprise and happiness (*p* < 0.001). Additionally, the accuracy in anger was also significantly lower than in happiness, and neutrality (*p* < 0.001). The differences between the emotions across calibration, SRC, and accuracy in ERA are illustrated in Figure 1.

## Discussion

The primary objective of this study was to examine how discrete emotion categories influence self-rated confidence (SRC), metacognitive calibration (MA), and emotion recognition accuracy (ERA) within a vocal context. As hypothesized, the one-way repeated-measures ANOVAs indicated significant differences among emotion categories for SRC, calibration, and ERA. However, the effect sizes were large for calibration and ERA, and small for SRC. Specifically, sadness elicited the highest calibration (significantly exceeding anger, fear, surprise, and happiness) and the highest ERA (significantly exceeding all other emotions), whereas fear showed the lowest calibration and among the lowest ERA. Moreover, pairwise comparisons for the SRC revealed no significant differences between specific emotions. In the following sections, we explore the nuances of these effects, beginning with an examination of MM and MA, and then turning to ERA.

### Metacognitive monitoring: SRC across emotions

While the main effect of emotion was significant, pairwise comparisons in the current study revealed no significant differences between specific emotions. This aligns with evidence across various modalities suggesting that emotion acts as a salient cue for metacognitive monitoring (MM). For instance, research on memory has shown that emotional valence serves as a primary driver for judgments of learning (Agadzhanyan and Castel, 2024). Similarly, in the tactile domain, positive and negative touch have been found to differentially modulate metacognitive judgments for emotional stimuli (Convertino et al., 2025). Within the vocal domain, our findings support the general consensus that emotional categories influence subjective MM (Lausen and Hammerschmidt, 2020). However, while the main effect of emotion was significant, pairwise comparisons in the current study revealed no significant differences between specific emotions. This finding is consistent with Bègue et al. (2019), who found no significant differences in SRC across stimulus intensities within facial ERA. However, previous studies in vocal prosody have identified distinct “confidence hierarchies,” where certain categories, such as anger and neutrality, elicit significantly higher SRC than others (Lausen and Hammerschmidt, 2020). The absence of such pairwise distinctions in our Hebrew sample may be attributable to methodological characteristics related to the stimulus set. Specifically, research by Troiano et al. (2021) suggests that SRC is strongly entangled with emotion intensity, where perceiving stronger affect prompts a more certain classification. Since the current study focused exclusively on the classification of discrete emotion categories and did not systematically manipulate or control for emotional intensity, it is possible that the diagnostic cues typically used by listeners to differentiate their SRC were obscured or lacked sufficient variability to trigger distinct pairwise differences.

Furthermore, the type of stimulus material appears to be a critical determinant of metacognitive certainty. Lausen and Hammerschmidt (2020) demonstrated that SRC is significantly higher for “affect bursts” (e.g., laughter or screams) compared to speech-embedded emotions, as the former provide more ecologically salient information. By using a single, repeated neutral sentence, our study relied on stimuli that may induce a familiarity effect. As noted in metacognitive research, item familiarity can lead to higher and more uniform confidence ratings, as participants may base their judgments on the familiarity of the lexical carrier rather than the subtle variations in prosodic cues. Finally, the significant main effect of emotion on SRC, without corresponding pairwise differences, may suggest that SRC is influenced by a combination of stimulus-related factors and individual difference variables. From this standpoint, previous research has demonstrated that trait-level characteristics such as empathy (Fischer et al., 2018) and metacognitive self-confidence (Kleitman et al., 2019) moderate the relationship between objective performance and subjective confidence across domains. Therefore, we recommend that future studies incorporate individual difference measures, including trait empathy, general metacognitive ability, and domain-specific self-efficacy, to better elucidate the underlying mechanisms of SRC in ERA.

### Metacognitive accuracy: calibration across emotions

While MM remained relatively stable across categories, a markedly different pattern emerged regarding MA, supporting an emotion-contingent calibration mechanism. Specifically, sadness exhibited the highest calibration scores (the smallest gap between SRC and ERA), while fear showed the poorest calibration. These findings suggest that although listeners may apply a similar SRC across various emotions, the validity of that confidence, its alignment with actual performance, is highly emotion dependent. Our data indicates that, for vocal emotions, MA is tightly linked to recognition performance, as sadness was both the most accurately recognized and the best-calibrated emotion, while fear showed low accuracy and poor calibration. However, the observed ERA results extend previous research in Hebrew vocal recognition (Sinvani and Sapir, 2022). Furthermore, our findings considered significant differences between emotions in calibration index align with prior evidence that MA is often influenced by task difficulty, even when subjective confidence remains high (Dentakos et al., 2019; Laukka et al., 2021).

Finally, our findings revealed that across all emotions, SRC levels were generally higher than ERA, indicating a general tendency toward overconfidence. This general pattern of overconfidence aligns with findings from Bègue et al. (2019) and Dentakos et al. (2019) who reported that participants overestimated their facial emotion recognition performance. This mirrors well-established patterns in social-perceptual tasks, in which averaged confidence exceeds actual performance (Stankov and Lee, 2014). However, the significant differences in calibration by emotion indicate that overconfidence is not uniform across categories. Such miscalibration may lead individuals to allocate fewer cognitive resources than needed in social interactions, potentially undermining performance (Binnendyk and Pennycook, 2024).

### Limitations and future directions

Although this study advances our understanding of MM and MA in vocal ERA, several limitations warrant consideration. First, we followed the discrete emotion framework, including six distinctive emotional expressions (Barrett, 2017). Future research should also incorporate additional approaches to facilitate the generalization of results and enable comparisons with previous cross-modal studies. Additionally, we believe that research would benefit from considering various types of stimuli, such as vocal bursts, words, and nonsense words, to better understand the nature of MA in emotion recognition across emotions (Lausen and Hammerschmidt, 2020). Furthermore, examining how different acoustic parameters (e.g., tone, intensity, speech rate) relate to confidence judgments could provide deeper insights into the cues that drive MM in vocal emotion recognition. Finally, future investigations should incorporate global measures, such as empathy questionnaires, alongside prospective judgments to further elucidate the mechanisms underlying MM and MA within the social domain.

## Conclusion

This study is among the first to systematically investigate how emotion category affects local metacognitive monitoring (MM) and metacognitive accuracy (MA) in a vocal emotion recognition task. Notably, we found similar variability trends in calibration and accuracy in ERA, suggesting that emotion-recognition accuracy may directly influence MA. We also observed a general tendency toward overconfidence across all emotions, though this was least pronounced for sadness. Our findings have important theoretical implications, supporting the view that miscalibration often manifests as overconfidence, which may hinder both immediate and future cognitive performance. Given the importance of MA in promoting social interactions and mental health, we recommend that future studies explore the underlying mechanisms of MM in emotion recognition and compare calibration indices across tasks and domains to provide a more comprehensive understanding of MA in social contexts.

## Data Availability

The data analyzed in this study is subject to the following licenses/restrictions: data is available upon request from authors. Requests to access these datasets should be directed to rachel-tzofia.sinvani@mail.huji.ac.il.
